# Changes in Novel AKI Biomarkers after Exercise. A Systematic Review

**DOI:** 10.3390/ijms21165673

**Published:** 2020-08-07

**Authors:** Wojciech Wołyniec, Wojciech Ratkowski, Joanna Renke, Marcin Renke

**Affiliations:** 1 Department of Occupational, Metabolic and Internal Diseases, Institute of Maritime and Tropical Medicine, Medical University of Gdańsk, 9b Powstania Styczniowego Street, 81-519 Gdynia, Poland; mrenke@gumed.edu.pl; 2 Department of Athletics, Gdańsk University of Physical Education and Sport, 1 Górskiego Street, 80-336 Gdańsk, Poland; maraton1954@o2.pl; 3 Department of General and Medical Biochemistry, University of Gdansk, 59 Wita Stwosza Street, 80-308 Gdańsk, Poland; joanna.renke@biol.ug.edu.pl

**Keywords:** urinary biomarkers, markers of AKI, cystatin-C, NGAL, KIM-1, exercise, acute kidney injury

## Abstract

More than 100 substances have been identified as biomarkers of acute kidney injury. These markers can help to diagnose acute kidney injury (AKI) in its early phase, when the creatinine level is not increased. The two markers most frequently studied in plasma and serum are cystatin C and neutrophil gelatinase-associated lipocalin (NGAL). The former is a marker of kidney function and the latter is a marker of kidney damage. Some other promising serum markers, such as osteopontin and netrin-1, have also been proposed and studied. The list of promising urinary markers is much longer and includes cystatin C, NGAL, kidney injury molecule-1 (KIM-1), liver-type fatty-acid-binding protein (L-FABP), interleukin 18, insulin-like growth factor binding protein 7 (IGFBP-7), tissue inhibitor of metalloproteinases-2 (TIMP-2) and many others. Although these markers are increased in urine for no longer than a few hours after nephrotoxic agent action, they are not widely used in clinical practice. Only combined IGFBP-7/TIMP-2 measurement was approved in some countries as a marker of AKI. Several studies have shown that the levels of urinary AKI biomarkers are increased after physical exercise. This systematic review focuses on studies concerning changes in new AKI biomarkers in healthy adults after single exercise. Twenty-seven papers were identified and analyzed in this review. The interpretation of results from different studies was difficult because of the variety of study groups, designs and methodology. The most convincing data concern cystatin C. There is evidence that cystatin C is a better indicator of glomerular filtration rate (GFR) in athletes after exercise than creatinine and also at rest in athletes with a lean mass lower or higher than average. Serum and plasma NGAL are increased after prolonged exercise, but the level also depends on inflammation and hypoxia; therefore, it seems that in physical exercise, it is too sensitive for AKI diagnosis. It may, however, help to diagnose subclinical kidney injury, e.g., in rhabdomyolysis. Urinary biomarkers are increased after many types of exercise. Increases in NGAL, KIM-1, cystatin-C, L-FABP and interleukin 18 are common, but the levels of most urinary AKI biomarkers decrease rapidly after exercise. The importance of this short-term increase in AKI biomarkers after exercise is doubtful. It is not clear if it is a sign of mild kidney injury or physiological metabolic adaptation to exercise.

## 1. Introduction

The analysis of human urine has been a part of medical practice for 6000 years. Uroscopy was “the mirror of medicine” or, in more ordinary terms, the first additional test in medicine, and was widely used to diagnose almost all medical conditions [1]. Now, urinalysis is one of the most common laboratory tests in medical practice.

Two-hundred years ago, the father of modern nephrology, Dr Richard Bright, discovered that patients with dropsy had albuminuria and structural changes in the kidneys. Dr Bright first described the classical nephrological triad and found a correlation between changes in urine (albuminuria) and diseased kidneys at autopsy [2]. Sixty-six years ago, Kenneth D. Gardner Jr. first described changes in urine after physical exercise. The proteinuria and hematuria were found in healthy subjects after relatively gentle exercise, therefore Gardner called these conditions “athletic pseudo-nephritis”, assuming that it is a physiological, transient and benign condition [3]. Those two observations defined the limits of our understanding of the significance of proteinuria. On the one hand, albuminuria is one of the most important markers of severe and sometimes fatal kidney diseases with well-described structural changes. But on the other hand, the list of physiological conditions in which transient proteinuria is observed is quite long. Protein in urine is found after exercise, exposure to cold or heat and protein-rich food (alimentary proteinuria), and proteinuria can also occur in pregnancy, fever, heart failure and in a vertical position (orthostatic, postural proteinuria) [4].

In recent decades, new methods of urine examination have been proposed: tubular enzymes, novel biomarkers of acute kidney injury (AKI), metabolomics, proteomics, transcriptomics and genomics [5,6,7]. The very promising new AKI biomarkers were called “kidney troponins” and hinted at the possibility of early diagnosis of kidney diseases. Some of the markers showed high sensitivity in AKI diagnosis. Numerous studies concerned urinary neutrophil gelatinase-associated lipocalin (NGAL), kidney injury molecule-1 (KIM-1), cystatin C (Cyst-C), liver-type fatty-acid-binding protein (L-FABP), interleukin 18, insulin-like growth factor binding protein 7 (IGFBP-7) and tissue inhibitor of metalloproteinases-2 (TIMP-2) [5,8,9]. Nevertheless, the only AKI biomarker test which is currently FDA (Food and Drug Administration)-approved for clinical use in the USA, and which is also used in some European countries, is NephroCheck, which combines TIMP-2 and IGFBP-7 [10].

The history of serum examination in kidney diseases is relatively short. In the last 100 years, creatinine established its position as the best marker of glomerular filtration rate (GFR) [11,12]. 22 years ago, Cyst-C was considered as an equal or even better marker of GFR than creatinine. Due to its higher price and lower availability, it is not widely used. Interestingly, although serum concentrations of both substances correlate strictly, they are eliminated by kidneys in two different ways. Both are freely filtered in the glomeruli, but creatinine is never reabsorbed and secreted, while cystatin C in healthy individuals is reabsorbed and metabolized in the proximal tubule. Therefore, in normal conditions, excretion of Cyst-C is very low [13]. Some other novel biomarkers of AKI, like NGAL and osteopontin, can be measured in serum [5].

The serum and urine markers of kidney injury were mainly studied in AKI. The aim of this review was to analyze changes of those markers after physiological condition—exercise. All but one of the studies analyzed were conducted in the last 10 years. The high number of proposed markers of AKI is sometimes confusing. Consequently, this review was ordered according to the classification suggested by Oh in a state-of-the-art review published this year (Table 1) [5]. The purpose of this review was to describe the newest markers of AKI, which is why conventional markers—creatinine, albuminuria, tubular enzymes—were not in the scope of the paper.

Repeated episodes of acute kidney failure may lead to chronic kidney disease (CKD); therefore, proper diagnosis of AKI is important [14]. There is no evidence that sport practicing can lead to chronic kidney problems; nevertheless, after marathon run and other endurance events, an acute renal failure requiring renal replacement therapy was observed [15]. The possible factors causing post-exercise AKI are dehydration, sub-clinical rhabdomyolysis, inflammation, increased energy demanding renal sodium uptake, reduced renal perfusion and nonsteroidal anti-inflammatory drugs (NSAIDs) frequently used by runners [16,17]. There is evidence that dehydration and soft drink intake during and following exercise may lead to acute kidney dysfunction [18] and that physical work in heat is leading to chronic kidney disease [19].

## 2. Results: Studies Concerning Novel Biomarkers of AKI after Exercise

### 2.1. Functional Biomarker—Serum Cystatin C

Cystatin C is a non-glucosylated 13 kD basic protein which belongs to the cysteine protein inhibitors family and is produced at a constant rate by all nucleated cells. Cystatin C is an inhibitor of lysosomal proteinases and one of the most important extracellular inhibitors of cysteine proteases [5,9]. Cystatin C is freely filtered in glomeruli and then reabsorbed and metabolized in the proximal tubule. Studies on diabetes, protein-induced glomerular hyperfiltration and extreme exercise demonstrated that acute changes in serum (s)Cyst-C provide a better approximation of GFR than serum creatinine (sCr). sCyst-C is affected by sex and race and to a small degree, by inflammation [20]. In clinical studies on acute kidney injury, an increase in serum and urine cystatin C levels is observed earlier than an increase in creatinine [5,9,20,21].

#### 2.1.1. Changes in sCyst-C after a Marathon

There were several studies dedicated to study changes in sCyst-C level after exercise. The increase in sCyst-C after a marathon was first noticed by Mingels et al. In this study of 70 recreational runners, the authors showed that the increase in sCyst-C is lower than the increase in sCr after exercise. This increase after a marathon was half that of creatinine (34% vs 53% increase, and after correction for the effect of dehydration, 21% vs 42%). Serum Cyst-C was increased above the upper reference limit in 46% of runners (in 26% after correction) [20]. Very similar changes—a significant increase in the sCyst-C level immediately after a marathon run—were observed by Scherr [22], McCullough [23] and Hewing [24] (Table 2).

The main differences in these studies concerned changes in the follow-up, but the time of the follow-up was defined in different ways. Therefore, it is difficult to compare those data. Nevertheless, all studies showed a rapid decrease in sCyst-C at rest.

#### 2.1.2. Changes in sCyst-C after Exercises Shorter than a Marathon

Poortmans et al. found that after a 30-min treadmill test at 80% of the maximal oxygen capacity, sCyst-C increased significantly by 13% (from 0.91 ± 0.06 to 1.03 ± 0.09 mg/L) and eGFR -Cyst-C decreased significantly by 19.8% [25]. Another study concerning subjects performing a submaximal test on a cycle ergometer at an exercise intensity of 80% of the maximal heart rate was performed by Bongers et al. In contrast to Poortmans, they did not find any changes in eGFR -Cyst-C after 30 min of exercise (eGFR 118 vs. 116 mL/min/1.73 m^2^), but after 150 min of exercise, a significant decrease in eGFR -Cyst-C to 103 mL/min/1.73 m^2^ was observed [26]. In Poortmans’ and Bongers’ studies, only males of the similar age (25 and 23 years) were studied. The difference between these two studies can be related to the type of exercise—in Poortmans’ study, a run on a treadmill, and in Bongers’ study, cycling on an ergometer [25,26].

#### 2.1.3. Changes in sCyst-C after Longer Exercise than a Marathon

Serum cystatin C was also measured after very long exercise—a 120 km “Infernal trail” race. Surprisingly, there was no change in sCyst-C (0.8 vs. 0.8 mg/L) and eGFR Cyst-C value even increased after the race—from 113.5 to 118.5 mL/min (*p* = 0.04). This could be due to the very low intensity of physical activity. The exercise was very long—a 120 km race with 5700 m positive elevation. The speed was very low (5.2 km/h) and the median time was 23.1 h [27].

#### 2.1.4. sCyst-C is a Better Marker of eGFR than sCr

Interesting observations were made in nine professional cyclists during the Giro d’Italia. In this study, blood was taken before, on the 12th and on the 22nd day of the race. The mean sCyst-C remained very stable: 0.61 ± 0.06 vs 0.62 ± 0.07 vs. 0.63 ± 0.06 mg/L. In this very interesting study, which is described in detail in two papers [28,29], blood was not taken immediately after the single race. Therefore, the study is not exactly in the scope of this review. Nevertheless, it provided evidence that even one of the most exhausting multistage efforts does not lead to an eGFR decrease in healthy, well-trained sportsmen [28,29]. Studies published by Banfi and Colombini showed that in athletes, sCyst-C is a better marker of eGFR than serum creatinine (sCr), also at rest. Some athletes, like cyclists, have a creatinine level that is lower than the reference values, due to a low lean mass (e.g., 9/9 cyclists taking part in the Giro d’Italia), while 12/15 professional rugby players had serum creatinine above the reference values due to their high body mass. In both of these studies, athletes had levels of serum Cyst-C in the normal range [28,29,30].

sCyst-C is also more precise than a sCr marker of eGFR in studies in which lean body mass is changing. In a study of a 6-month physical activity program in obese boys, serum creatinine increased, but sCyst-C remained unchanged. In the subjects, the lean mass and height increased, while their weight did not change [31].

#### 2.1.5. Summary of Changes in sCyst-C

In summary, the main advantages of sCyst-C over creatinine in studies concerning exercise is that sCyst-C is not correlated with lean mass [28,29,30,32]. Therefore, sCyst-C may be more suitable for assessing renal function in individuals with a higher muscle mass when mild kidney impairment is suspected [33]. The studies performed after single exercise may suggest that sCyst-C elevation is dependent on intensity and duration. Long and intensive exercises such as a marathon will cause an increase [20,22,23,24], while short exercises or exercises with lower intensity will not [26,27].

### 2.2. Plasma and Serum Damage Markers

Damage markers can help in early AKI diagnosis even before elevation of sCr and sCyst-C levels [5]. Only a few damage markers are measured in serum or plasma: NGAL, KIM-1, osteopontin and netrin-1. The most studies concerned changes in NGAL.

NGAL, also known as siderocalin or lipocalin 2, is a member of the lipocalin superfamily of carrier proteins, which are approximately 25 kDa in size. NGAL has a bacteriostatic function related to its ability to bind iron-siderophore complexes and thereby prevents iron uptake by bacteria. NGAL also provides an antiapoptotic effect and enhances proliferation of renal tubular cells [8]. It is produced by activated neutrophils in the proximal tubules. NGAL is filtered in the glomerulus and reabsorbed in the proximal tubule. After ischemic, septic or toxic kidney injury, NGAL is dramatically upregulated at the transcript and protein level. Plasma and urinary NGAL levels are significantly increased in those with early structural renal tubular damage caused by various factors [5,8,9].

#### 2.2.1. Changes in Plasma NGAL (pNGAL) after Short Exercises

Changes in pNGAL after exercise were first investigated by Junglee et al. in 2012. In this study dedicated to AKI in exercise, after relatively short exercise (an 800 m run), pNGAL was decreased, which was interpreted by the authors as an effect of increased NGAL renal clearance [34].

In another study performed by Junglee, the pNGAL level increased after a 40-min heat stress run (running on a treadmill on a 1% gradient for 40 min at 65% VO_2max_ (maximal oxygen consumption) in an environmental chamber maintained at a dry bulb temperature of 33 °C with 50% relative humidity (RH)). In this study, the heat stress run was preceded by a 60-min downhill muscle-damaging run (EIMD group) or a 60-min flat run (CON group) and a 30-min seated rest. pNGAL increased in both groups, but the increase was greater in the EIMD group [35].

There were also three studies dedicated to investigating a pNGAL as a marker of inflammation, neutrophil degranulation and organ damage, but not an AKI biomarker [36,37,38].

Bender et al. studied pNGAL as an inflammatory marker of hand osteoarthiritis (OA) after mechanical exercise of the OA hand. pNGLA increased during the first 15 min after exercising the index hand within the venous blood of the ipsilateral forearm [36]. Kanda et al. studied 9 untrained men during a one leg, calf-rise exercise. pNGAL was studied as a marker of organ damage, muscle disruption and neutrophil mobilization and migration. The authors did not find any changes in pNGAL [37]. Rullman et al., found no significant changes in pNGAL after 27 and 57 min of cycle exercise. During the first 20 min, the subjects exercised at 50% of VO_2max_ and during the next 40 min, at 65% VO_2max_. In the Rullman study, pNGAL was investigated as a marker of neutrophil degranulation [38].

#### 2.2.2. Changes in pNGAL or Serum NGAL (sNGAL) Levels after Long Exercises

Chapman et al. studied the impact of soft drink consumption during long exercise in heat. Twelve healthy subjects drank two liters of a beverage (soft drink or water) during four hours of exercise in 35.1 °C heat. pNGAL increased post-exercise in both groups [18].

McDermott et al. found a 2-fold significant increase in sNGAL (from 68.51 to 139.12 ng/mL) after a 6-h endurance cycling event during heat (33.2 ± 5.0 °C, 38.4 ± 10.7% RH). Moderate ibuprofen ingestion of 600 mg ibuprofen had no influence on the sNGAL level [39]. Moreover, Lippi et al., found a significant 1.6-fold increase in sNGAL (from 105 to 196 ng/mL) after a 60-km run in a group of trained male athletes [40].

Furthermore, Andrezzoli et al. found only a mild increase in pNGAL in a group of professional cyclists after the mountain stages of two major European professional cycling competitions (Giro D’Italia and Tour de France). Post-competition NGAL values of all the variables investigated remained within the physiological range. The results suggest that even if NGAL values rose slightly and not significantly after competition, no kidney injury occurred in these highly trained athletes during the mountain stages of professional competitions [41].

NGAL, which is an acute phase protein and is upregulated in the lungs during inflammation, was also studied as a marker of inflammation and oxidative stress after long exercise. Mellor et al. found a non-significant NGAL rise after an ascent from sea level to 1085 m over 6 h [42]. In this study, two other cohorts were also studied. There were no changes in NGAL after 3 h exposure to normobaric hypoxia with a 5-min step test, but there was an increase in NGAL after trekking in Nepal [42].

#### 2.2.3. Changes in pNGAL after Work in Heat

Chapman et al. analyzed changes in pNGAL and other biomarkers in two interesting studies. In the first, the impact of different beverage consumption (soft drink or water) during exercise in heat was studied. Twelve healthy subjects drank two liters of fluid during four hours of exercise in 35.1 °C heat [18]. In the second study, thirteen healthy adults (3 women, 10 men, age 23  ±  2 years) exercised for 2 h in a 39.7  ±  0.6 °C, 32%  ±  3% relative humidity environmental chamber. In four trials, the subjects received water to remain hydrated (*Water group*), were exposed to continuous upper-body cooling (*Cooling group*), a combination of both (*Water + Cooling group*), or no intervention (*Control group*) [43]. In the first study, in both groups, pNGAL was increased post-exercise and returned to pre-exercise levels after 24 h [18]. In the second study, an increase in pNGAL was also observed and was greater in the control group (without hydration and cooling) compared with the other conditions [43].

#### 2.2.4. Summary of Changes in s/pNGAL

The importance of s/pNGAL in the diagnosis of AKI in exercise is questionable. NGAL is released by respiratory epithelium, liver and heart, and therefore changes in the s/pNGAL level could be caused by inflammation, hypoxia or muscle damage, conditions which are integral to exercise [35,42]. Therefore, it is unclear to which degree, if any, an increase in NGAL after exercise is related to kidney injury. The methodological problem is that a huge difference between athletes is observed [40].

### 2.3. Urinary Damage Markers

#### 2.3.1. Urinary Cystatin C (uCyst-C)

Since Cyst-C is freely filtered by the glomerulus, reabsorbed and metabolized in the renal tubule, even a small elevation of urinary Cyst-C (uCyst-C) reflects proximal tubule injury [13].

Bongers et al. studied subjects performing submaximal exercise at an 80% HR rate and found a significant increase in uCyst-C with higher values after prolonged exercise (150 min) compared to acute (30 min) exercise [26]. In 2012, the same authors studied urinary markers after single and repetitive bouts of exercise. They examined participants of the International Four Day Marches Nijmegen. Subjects walked at 70% intensity over 30, 40 or 50 km for 3 consecutive days. Bongers studied several urinary markers and found that uCyst-C increased 1.8 times after the first day (from 0.05 to 0.09 mg/L), but this effect disappeared on day 3 (uCyst-C = 0.06 mg/L) [44]. Interestingly, in these studies, uCyst-C was measured mainly to normalize uKIM-1 and uNGAL levels [26,44]. The increase in uCyst-C was also found by Wolyniec after 10 and 100 km runs. There was a 2.56-fold increase after 10 km and a 4.96-fold increase after 100 km. When normalized to creatinine, these increases were 1.39- and 1.95-fold, respectively [45].

The number of studies coming from only two centers is small, but it seems that uCyst-C is a very sensitive marker of proximal tubule dysfunction after exercise.

#### 2.3.2. Changes in uNGAL and uKIM-1 after a Marathon

KIM-1 is a 38.7 kDa type 1 transmembrane glycoprotein member of the TIM family of immunoglobulin superfamily molecules. KIM-1 plays a role in kidney recovery and tubular regeneration because it acts as a phosphatidylserine receptor and thereby mediates the phagocytosis of apoptotic cells. KIM-1 protects kidney against ischemic-reperfusion injury [8]. KIM-1 was found to be expressed at low to undetectable levels in normal kidney tissue but is markedly expressed after ischemic or toxic injury in proximal tubule cells. KIM-1 can serve as a urine and blood AKI biomarker. KIM is elevated in early stages of AKI and its urinary concentration is closely related to the severity of renal damage [5,8,9].

The first study concerning changes in urinary NGAL and KIM-1 after a marathon was performed by McCullough and published in 2010 [23]. The authors showed a 5.7-fold increase in uNGAL and a minor rise in uKIM-1 after a marathon [23]. According to the authors, those were changes “supporting a pathobiologic case for AKI” [23]. Changes in uKIM-1 and uNGAL levels after a marathon were also studied by Mansour et al. [46] The results concerning uNGAL were very similar to these from McCullough’s study (a 4.71-fold increase in uNGAL), but the increase in uKIM-1 was much higher. The decrease in uKIM-1 was slower than the other markers studied (uNGAL, uTNF-alfa [tumor necrosis factor α], uIL-18, uIL6, uIL8, uYKL-40, uMCP-1) and 24 h after a marathon, the level was still increased (Table 3) [46].

#### 2.3.3. Changes in uNGAL after Exercises Shorter than a Marathon

No changes were found in the uNGAL level in Kanda’s study on 9 untrained males during a one leg calf-rise exercise [37] and in the Wołyniec study of amateur runners after a submaximal test on a treadmill [47]. In contrast, in two other studies, uNGAL was increased after very short exercise. Junglee et al. noticed an increase in uNGAL and uNGAL/uCr immediately and 25 min after an 800 m run. The uNGAL level returned to the baseline levels after two hours [34]. Spada et al. also noticed an increase in uNGAL after 4 min of an high-intensity interval resistance training (HIIT) session (eight sets of squats performed with the fastest speed and the highest number of repetitions achievable in 20 s with 10 s of rest between sets). In this study, uNGAL was increased in women 2 after exercise and returned to values similar to the baseline 24 h after exercise. In 5/29 females, uNGAL/uCr exceeded 100 ng/mgCr, the value of which is compatible with clinical AKI. In men, the increase in uNGAL and uNGAL/uCr was not statistically significant [48].

Junglee et al. found an 8-fold uNGAL increase after a 40-min heat stress run (65% VO_2max,_ 33 °C): 80% of subjects from the muscle-damaging group and 30% from the flat-run group had uNGAL above the normal range after exercise [35]. Bongers, who studied uNGAL after 30 and 150 min of exercise, found that uncorrected uNGAL and uNGAL corrected to osmolality were increased, while there were no changes in uNGAL corrected to creatinine and cystatin-C [26]. After a 10-km run, both uNGAL and uNGAL/Cr increased significantly (3.9- and 2.9-fold, respectively) in the Wołyniec study [45]. Otherwise, in Semen et al.’s study, a 10 km run caused an increase in uNGAL only when combined with ibuprofen/naproxen use [49]. In the same study, a significant increase in uNGAL was observed in the half-marathon runners [49].

In another study, Semen et al. found that completion of a half marathon after use of a 400 mg single dose ibuprofen led to a 2-fold increase in uNGAL. However, this increase was smaller and not significant in the group supplemented with monomeric and oligomeric flavanols (MOF-VVPP) [50]. In the Wolyniec study, the increase in uNGAL was higher than in Semen’s study, although the exercise was shorter. This difference could be partially explained by the higher intensity of a 10-km run but could also be related to the methodology. In the first study, urine samples were collected immediately after the run and in the second, urine samples were collected within 2 h after the run [45,50].

#### 2.3.4. Changes in uNGAL after Exercises Longer than a Marathon

The uNGAL level was elevated after all exercises longer than a marathon run. Bongers found that prolonged walking exercise at 70% intensity caused a 2.25-fold increase in uNGAL on day 1, and a 1.54-fold increase on day 3 compared to the baseline levels [44]. An uNGAL increase was also found by Lippi et al. after a 60-km run (7.7- fold increase in uNGAL) [40], by Jouffroy et al. after an 80-km run (5-fold increase after 53 km, and 2.5-fold after 80 km, without significant changes in uNGAL/uCr) [51], by Wolyniec et al. after 100 km (6.82-fold increase in uNGAL, and only a 2.94-fold increase in uNGAL/uCr) [45] and by Poussel el al. after a 120-km run (2.6-fold increase in uNGAL and a 1.5-fold in uNGAL/uCr) [27]. Only 6.25% of the participants in the Wołyniec study and 12.5 % in the Poussel study had uNGAL/uCr above the reference value [27,45].

#### 2.3.5. Changes in uNGAL after Exercise in Heat

Chapmen et al., performed two exciting studies, as mentioned above [18,43]. In the first, they found that 24 h after exercise in heat, uNGAL was elevated above the pre-exercise level in subjects drinking soft drinks, although uNGAL corrected to uCr osmolality did not produce any changes [18]. In the second study, uNGAL was elevated after 2 h of exercise in heat, and this increase was greater in the control group compared with the other conditions (hydration or/and cooling) [43].

#### 2.3.6. Summary of Changes in uNGAL

Changes in uNGAL were typically found after long exercise. It seems that uNGAL is frequently increased, but rarely exceeds normal values when normalized to creatinine. Some factors, like environmental temperature, type of cooling and hydration, are related to changes in uNGAL. Interpreting these changes is difficult. Machado found elevated levels of uNGAL in endurance cycling athletes 48 h after exercise and suggested that the increase in uNGAL is related to metabolic adaptation to endurance exercise, or possibly predisposition to acute kidney injury over time [52]. In Bongers’ study, uNGAL was elevated after the first day of marching and then decreased, which also suggested some kind of kidney adaptation to exercise [44].

#### 2.3.7. Changes in Urinary KIM

Except for the two studies after a marathon mentioned above [23,46], uKIM was studied only in 5 studies coming from 3 centers. In all these studies, uKIM-1 was increased.

Bongers et al. found an increase in uKIM-1 after 30- and 150-min submaximal exercise [26] and after one day of walking, with a subsequent decrease in its level after 3 consecutive days of marching [44]. In the first study, the uKIM-1 corrected to uCr, uCyst-C and urine osmolality showed no significant changes [26]. In the second study, uKIM- 1 corrected to osmolality was increased, while uKIM-1/uCr and uKIM/uCyst-C ratios were unchanged [44]. Wolyniec found an increase in uKIM-1 but not in the uKIM/uCr ratio after a treadmill test, 10 and 100 km runs [45,47]. Jouffroy found a significant increase in uKIM-1 but not in uKIM/uCr during an 80-km run. Interestingly, nine days after the race, uKIM-1 remained significantly higher than the baseline level [51].

#### 2.3.8. Summary of Changes in uKIM-1

uKIM-1 was increased after all exercises, but when normalized to uCr, it was unchanged. The changes in uKIM-1 were long-lasting, uKIM-1 was elevated 2 days after a marathon [46] and 9 days after an 80 km run [51]. At the same time, uNGAL decreased more rapidly.

#### 2.3.9. Changes in Urinary L-FABP after Exercise

L-FABP belongs to the fatty acid-binding protein superfamily and has a molecular mass of about 14 kDa. The function of the members of the FABP family is the regulation of fatty acids uptake and the intracellular transport. L-FABP binds fatty acids and transports them to the mitochondria and peroxisomes. L-FABP also protects renal cells from oxidative stress [8]. The urinary L-FABP level is correlated with the peritubular capillary flow and ischemia. It appears to be a promising biomarker for both the diagnosis and prediction of AKI and its outcomes among critically ill patients [5,8,9]. L-FABP is localized in the proximal tubule and secreted into urine in response to a number of different intrarenal stresses, such as proteinuria, hypoxia, hyperglycemia, hypertension and oxidative stress [37,53,54].

Only two studies in healthy populations concerning changes in uL-FABP after exercise have been published. uL-FABP was significantly increased after incremental short maximal exercise on a cycling ergometer in a group of 116 adults of variable age (24–83 years) in a study published by Kosaki et al. In this experiment, uL-FABP/uCr changes were independently correlated with albuminuria, which supported previous observations that protein overload in the proximal tubule may cause an increase in uL-FABP [53]. After short exercise (one leg calf-rise exercise), Kanda et al. did not find any changes in uL-FABP [37].

#### 2.3.10. Other Studies Concerning Changes in uL-FABP

Hiraki showed that after a single case of a 20-min moderate intensity exercise (20-min treadmill walking, 40–60% exercise intensity) session in 31 adults with chronic kidney disease (CKD), there was no change in uL-FABP. This exercise was rather gentle and even albuminuria was not increased [55]. Kosaki studied individuals aged 50–83 without CKD and found that uL-FABP was the lowest in participants with a higher level of aerobic fitness and muscular strength [56,57] and that 12-week aerobic exercise training significantly decreases uL-FABP levels [57]. Relative changes in uFABP were significantly correlated with the relative changes in physical activity and the mean arterial pressure after intervention. The authors concluded that “habitual exercise appears to be associated with the degree of several stresses on the proximal tubule and to be beneficial for kidney health in middle-aged and older adults” [57]. Uchiyama et al. found a decrease in uL-FABP after a 12-week, home-based exercise program involving 47 patients undergoing peritoneal dialysis [58].

#### 2.3.11. Urinary Interleukins

Urinary interleukins, Il-1, Il-6, IL-8 and Il-18, were proposed as markers of AKI. Interleukins are important mediators of the immune reaction in the innate immune system response and adaptive immunity [8]. All these cytokines are freely filtered and then reabsorbed and metabolized in the proximal tubule; therefore, tubular injury leads to an elevation in their levels in urine [5,6,8,9,48].

Manosur et al. studied changes in urinary interleukins after a marathon and found a 19.2-fold increase in uIL-6, a 9.13-fold increase in uIL-8 and a 7.13-fold increase in uIL-18 [46]. Similarly, Semen et al. observed significant increases of urinary interleukins after a half marathon and the use of 400 mg ibuprofen. There was a 10-fold increase in uIL-6, a 2.87-fold increase in uIL-8 and a non-significant increase in IL-18. The elevations of uIL-6 and uIL-8 were smaller in runners supplemented with MOF-VVPP (5.8- and 1.49-fold, respectively) [50]. Elevation of uIL-6 and uIL-8 was also found by Sugama et al. after a duathlon [59]. Spada et al. found an increase in uIL-18 after a HIIT session [48]. Dutheil et al. found an increase in uIL-8 after a 24 h work shift and, according to the author, this elevation was related to stress [60].

### 2.4. Pre-Injury Phase Biomarkers IGFBP-7/TIMP-2

Insulin-like growth factor binding protein 7 (IGFBP-7) is a 29 kDa protein, a member of IGFBPs. It is a kind of glycoprotein with a molecular weight of 30 kDa. IGFBP-7 is known to bind and inhibit signaling through IGF-1 receptors [8]. Urinary IGFBP-7 is increased in kidney damage caused by sepsis or ischemia [8,9].

TIMP-2 is a 21 kDa protein, a member of the TIMP family. TIMP2 is a member of the tissue inhibitor of matrix metalloproteinase family. TIMP2 is an endogenous inhibitor of metalloproteinase activities and participates in the regulation of cell growth and apoptosis [8,9].

Combined urinary IGFBP-7 and TIMP-2 predict the occurrence of AKI better than other markers (NGAL KIM, IL19) [8,9]. NephroCheck, which combines TIMP-2 and IGFBP-7, is the only FDA-approved AKI biomarker test for use in the USA and is also used in some European countries [10].

Surprisingly, IGFBP-7 and TIMP-2 were studied only in one study after exercise. Chapman et al. studied thirteen healthy adults exercising in heat (the study is described above) and found elevated levels of uIGFBP-7 and uTIMP-2. There was a greater increase in the urinary biomarkers of AKI in the *Control* group. The differential findings between IGFBP7 (preferentially secreted in the proximal tubules) and TIMP-2 (secreted in the distal tubules) suggested that the proximal tubules are the location of potential renal injury [43].

### 2.5. Other Promising Markers of AKI (YKL-40, MCP-1 and TNF-alfa, Trefoil Factor 3 (TTF3), Calbindin)

There are over 100 biomarkers of AKI [61]. The urinary biomarkers which have been assessed in numerous studies are: chitinase 3-like protein 1 (YKL-40), MCP-1, TNF-alfa, osteopontin, DKK-1, micro RNAs, hemojuvelin, clusterin, CYR-61, cytochrome-C, epidermal growth factor, malondialdehyde, calprotectin, urine AGT angiotensinogen, matrix metalloproteinase 9, urine cysteine-rich 61, Na^+^/H^+^ exchanger isoform 3 protein, netrin-1, fetuin-A and trefoil factor 3 (TFF3) [5,8,9]. Most of these markers were not studied after exercise.

In one of the most interesting and largest studies concerning AKI biomarkers after exercise, Mansour et al. found increases in several urinary markers: a 4.5-fold increase in TNFα, a 6.69-fold increase in MCP-1 and an 8.99-fold increase in YKL-40 after a marathon [46]. Sugama et al. found increased uMCP-1 after a duathlon, but this change was not significant when normalized to uCr [59]. Semen et al. found a significant uTNFα increase after a half marathon [50]. Calbindin and TTF3 were studied in one study, and both of these markers increased after HIIT training [48]. The TTF3 family is a group of small molecule polypeptides, and uTTF3 was significantly reduced following renal tubular damage [9]; therefore, the increase in uTTF3 after exercise was surprising.

## 3. Discussion

Studies concerning new markers of AKI after physical exercise combine two different entities. Markers of AKI were introduced to diagnose the early phase of kidney injury in critically ill patients with sepsis or shock [5,6,8,9], but in these studies, they were measured in healthy subjects during physical activity, like walking, running or cycling. The increase in new AKI biomarkers was anticipated in these studies, because even much less sensitive markers, like serum creatinine and urinalysis, show changes after exercise [3,23].

Although many markers of AKI were described, only a minority were studied after exercise. Some markers are classified as injury markers (e.g., uKIM-1, uTNFalfa, uIL-6, uIL-8, uIL-18, uNGAL) and others as repair biomarkers (e.g., uYKL-40, uMCP-1) [46]. Another classification is based on the site of injury. There are markers of tubular (e.g., NGAL, IL-18, L-FABP, KIM-1, IGFBP-7) and glomerular (e.g., matrix metallopeptidase 9 [MMP-9]) injury [9]. The authors of this review used the classification proposed by Oh et al. [5] (Table 1), which has practical implications.

### 3.1. Limitations of the Studies Presented

**Figure 1:**
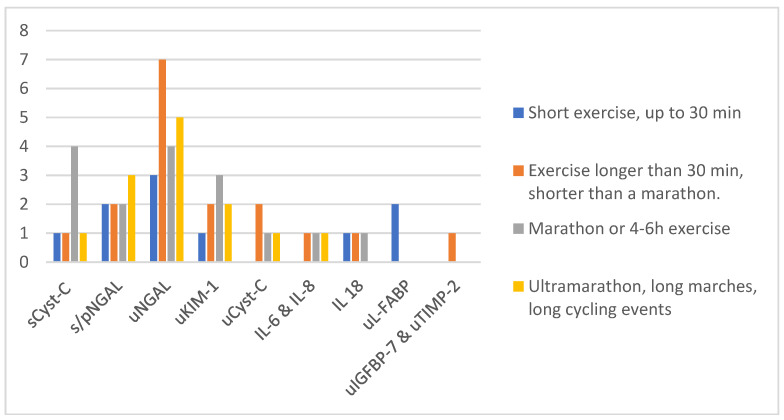
Number of studies in different types of exercise. Abbreviations: u—urinary, s—serum, p—plasma, Cyst-C—cystatin C, NGAL—neutrophil gelatinase-associated lipocalin, KIM-1—kidney injury molecule-1, L-FABP—liver-type fatty-acid-binding protein, IL—interleukin, IGFBP-7—insulin-like growth factor binding protein 7, TIMP-2—tissue inhibitor of metalloproteinases-2.

There are several limitations to the studies presented in this review. Twenty-seven studies were analyzed. All but one were published during the last 10 years and 15/27 in the last 5 years. The number of subjects studied ranged from 9 to 167, although most of the studies had a small number of participants: in 16 studies, fewer than 30 participants were investigated and only in 3 studies were there more than 100 participants studied. In 12 studies, only males were analyzed, and only in 7 studies were both sexes represented to the same or very similar degree. In three studies, all concerning marathons, more females were analyzed. The mean age of the participants differed greatly, ranging from 20 to 60 years. There were many different study designs proposed by the researchers, although the most common was a marathon (distance 42,195 m), which was used in 5 studies (Figure 1, Table 4).

The diagnosis of AKI was never confirmed by kidney biopsy, which is completely understandable. The studies were performed in relatively small groups and no cases of AKI requiring HD or rhabdomyolysis were observed, because both of these severe complications are extremely rare after exercise. There is insufficient data to describe changes in AKI biomarkers in such severe complications of exercise.

The follow-up was defined in a different way in the studies presented, and in some, there was no follow-up at all.

Suggestions: Studies in larger groups, preferably multicenter, are needed. It could be reasonable to study all markers after a marathon, which is the classical distance, relatively long and intensive. Indeed, it is the most commonly studied type of exercise. More studies in females are also required. Studies with a precisely defined follow-up with several time points, as well as observational case studies on changes in AKI biomarkers in subjects with severe complications could be very interesting.

### 3.2. Serum or Plasma Markers

Reasonable data only concern cystatin C and NGAL. The results of the studies presented provided enough information to consider cystatin C as a better marker of eGFR than creatinine after exercise and at rest in athletes with high or low lean mass [28,29,30]. The information concerning plasma or serum NGAL is more questionable. In fact, after some exercises, NGAL is elevated. However, NGAL is also a marker of inflammation, organ damage and hypoxia, and in exercise, it seems to have low specificity for AKI. One practical problem is the huge variability of levels between studies and subjects. One of the possible implications of p/sNGAL measurement is diagnosis of subclinical AKI in uncomplicated rhabdomyolysis, when creatinine and cystatin levels are within the normal range [62].

The general problem with serum measurement after exercise is hemoconcentration. In many publications, the authors used a correction of the effect of dehydration according to Dill and Costill’s method [63], on the basis of changes in pre- and post-blood morphology. This approach is reasonable in experimental studies, but in clinical practice, it is difficult to use, because pre-injury blood morphology results are unknown.

Suggestion: We suggest measuring sCyst-C instead of creatinine in future studies of kidney function in exercise. It is reasonable to check sNGAL in the risk group with rhabdomyolysis.

### 3.3. Urinary Markers

The urinary markers are increased after almost every exercise. The increment is rather small but consistent with individuals. The changes are dependent on the duration and intensity of exercise. Most studies investigated changes in uKIM-1 and uNGAL. After short exercise, an increase in uKIM-1, but not uNGAL, was observed. Elevated uKIM-1 was observed 2 and even 9 days after prolonged exercise. It is difficult to discuss the utility of uL-FABP, uCyst-C and other markers, because only few studies were performed. L-FABP is a marker of hypoxia, therefore it could be an ideal marker for studies in exercise but was used only in very few studies from one study group. What is also surprising is that uIGFBP-7 and uTIMP-2 were only analyzed in one study, and were the only markers approved for early diagnosis of AKI. The methodological problem with interpreting the changes in urinary markers is normalization. It is known that all urinary markers can be diluted, and, e.g., normalization of albuminuria is a standard procedure. In some studies, un-normalized values are used, but most authors normalized AKI markers to creatinine, osmolality, urine flow or cystatin C [18,23,26,27,34,35,40,44,45,46,47,48,51,53]. All these approaches had some limits. The most common was normalization to creatinine.

Suggestion: There is a need for studies on follow-up. Studies showing changes in urine markers shortly after exercise are interesting but have little practical value. In clinical practice, AKI is suspected and diagnosed several hours after exercise. What is most important is what levels of markers are typical for AKI 3, 6, 12 or 24 h after exercise. Although normalization to creatinine has some limits, it is the most common approach, and therefore it is reasonable to use this kind of normalization in subsequent studies.

There is no biomarker specific enough to assess AKI as a single biomarker. There is also no panel assessment using a couple of biomarkers, except combined urinary IGFBP-7 and TIMP-2. Taking into account the results presented in this review, combined uKIM-1/uCr and uNGAL/uCr could be the best to exclude or diagnose AKI after exercise.

### 3.4. Interpretation

In the presented studies, changes in AKI biomarkers were common. The main problem is how to implement the knowledge from these studies in clinical practice. There are several facts concerning AKI biomarkers, AKI and CKD in athletes:

1. Exercise-induced renal impairment is commonly present but temporary,
2. Severe complications of exercise, like AKI requiring hemodialysis, are rare,
3. Repeated episodes of AKI lead to CKD,
4. There is no data showing that CKD could be related to sports activity,
5. There is a growing body of evidence that exhausting work in heat leads to CKD [19,58,59,60,61,62,63,64] and the same risk factors—dehydration and muscle damage, soft drink consumption—are present, for example, in marathon runners.

Suggestion: We suggest that further studies on the physiological role of biomarkers are needed. Epidemiological studies and studies on athletes who have completed several dozen marathons or other long forms of exercise and studies on the impact of work and soft drinks on kidney function are awaited.

## 4. Materials and Methods

**Figure 2:**
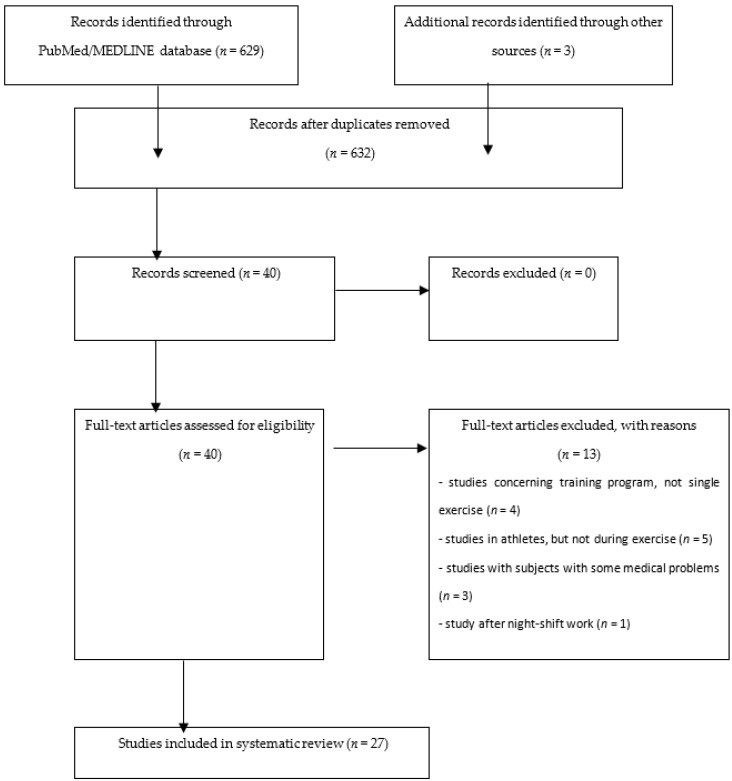
Flow chart illustrating the procedure for article inclusion and exclusion in a systematic review of changes in AKI biomarkers after exercise.

The authors researched the PubMed/MEDLINE electronic database by using terms consisting of the following: (AKI biomarker or cystatin C or NGAL or KIM-1 or urinary interleukin-18 or urinary interleukin or urinary liver-type fatty acid-binding protein or urinary L-FABP or urinary insulin-like growth factor-binding protein 7 or urinary IGFBP7 or urinary tissue inhibitor of metalloproteinases-2 or urinary TIMP-2 or nephrocheck or urinary osteopontin or urinary calbindin or urinary TTF) (nordic walking or physical activity or exercise or marathon or ultramarathon or swimming or cycling or games or football). The search was repeated regularly, and the database was updated until the last update on 13 July 2020, prior to manuscript submission. An additional search was conducted according to a reference list of read papers and by using investigators’ names. As the initial selection was done through titles and abstracts, it is possible that some important papers might have been omitted, although the authors tried to identify all that were important. A total of 27 papers were retrieved from 629 titles and abstracts identified in the PubMed database (Figure 2).

In this review, results of 13 additional studies were also shortly discussed. These papers did not consider single exercise in healthy subjects, but revealed important information, and the authors decided to present their results. There was no possibility to perform any statistical analysis because of the high variability of schedules, experimental conditions and different study groups described in the papers cited. Ethical approval was not necessary, because the study did not involve participants.

## Figures and Tables

**Table 1:** Biomarkers of acute kidney injury (AKI) studied in exercise discussed in this review—classification according to Oh et al. [5].

Functional Biomarkers	Damage Biomarkers	Pre-Injury Phase Biomarkers
sCyst-C	uCyst-C uNGAL, sNGAL, pNGAL uKIM-1 uL-FABP uIL-6, uIL-8, uIL-18, uTTF uCalbindin uTNFα uYKL-40 uMCP-1	uIGFBP-7 uTIMP-2

**Abbreviations:** u—urinary, s—serum, p—plasma, Cyst-C—cystatin C, NGAL—neutrophil gelatinase-associated lipocalin, KIM-1—kidney injury molecule-1, L-FABP—liver-type fatty-acid-binding protein, IL—interleukin, TTF3—trefoil factor-3, TNF*α*—tumor necrosis factor α, YKL-40—chitinase 3-like protein 1, MCP-1—monocyte chemoattractant protein-1, IGFBP-7—insulin-like growth factor binding protein 7, TIMP-2—tissue inhibitor of metalloproteinases-2.

**Table 2:** Changes in sCyst-C level after a marathon.

Study	sCyst-C Before a Marathon (mg/L)	sCyst-C after a Marathon (mg/L)	The Relative Increase in sCyst-C (%)	sCyst-C in Follow-Up (mg/L)
Mingels et al. [20]	0.71 (0.56–0.95)	0.95 (0.63–1.45)	34% (21% after correction of effect of dehydration)	0.73 (0.6–0.93) (day after, measured only in 18/70 subjects)
Scherr et al. [22]	0.77 (0.71–0.85)	0.94 (0.86–1.01)	22%	0.9 (0.81–1.00) (24 h after the race) 0.81 (0.72–0.86) (72 h after the race)
McCullough et al. [23]	0.8 ± 0.1	1.0 ± 0.2	25%	0.8 ± 0.1 (24 h after the race)
Hewing et al. [24]	0.68 (0.75–0.93)	0.85 (0.69–0.99)	25%	0.66 (0.59–0.78) (14 days after the race)

**Abbreviations:** sCyst-C—serum cystatin-C.

**Table 3:** Changes in uNGAL and uKIM-1 after a marathon.

Study	uNGAL before a Marathon (ng/mL)	uNGAL after a Marathon	Fold Increase	KIM-1 before a Marathon	uKIM-1 after a Marathon	Fold Increase
McCullough et al. [23]	8.2 ± 4.0	47.0 ± 28.6 (10.6 ± 7.2 after 24 h)	5.73× (1.29×)	2.6 ± 1.6 ng/mL	3.5 ± 1.6 (2.7 ± 1.6 after 24 h) ng/mL	1.35× (1.03×)
Mansour et al. [46]	8.00 (4.15–30.48)	37.64 (19.03–84.61) (day 2: 18.49 (9.25–33.69))	4.71× (2.31×)	132.59 (67.61–219.98) pg/mL	723.32 (459.36–1970.64) (day 2: 702.42 (123.27–1098.67)) pg/mL	5.46× (5.3×)

**Abbreviations:** uNGAL—urinary neutrophil gelatinase-associated lipocalin, uKIM-1—urinary kidney injury molecule-1.

**Table 4:** Studies on changes in new AKI markers after single exercise in healthy subjects—ordered according to the year of publication.

Author (Year of Publication)	Study Group	Exercise/Study Design	Markers
Mingels et al. (2009) [20]	70 recreational male runners age 47 (range 30–68) years	marathon	sCyst-C
Scherr et al. (2011) [22]	102 healthy male runners age 42 ± 9.5 y	Marathon	sCyst-C
McCullough et al. (2011) [23]	25 healthy runners age 38.7 ± 9.0 years (13 females, 12 males)	Marathon	sCyst-C uNGAL uKIM-1
Poortmans et al. (2012) [25]	12 male physical educators age 25 ± 5 years	30-min treadmill exercise at 80% of VO_2_max	sCyst-C
Junglee et al. (2012) [34]	20 healthy active adults age 24 ± 4 years (7 females, 13 males)	800-m sprint	pNGAL uNGAL uNGAL/uCr
Rullman et al. (2012) [38]	10 healthy men age 25 (range 18–37) years	60-min cycle ergometer test (20 min at 50% of VO_2_max; +40 min at 65% of VO_2_max)	pNGAL
Lippi et al. (2012) [40]	16 trained male athletes age 42 (range 34–52) years	60-km ultramarathon	sNGAL, uNGAL, uNGAl/uCr
Junglee et al. (2013) [35]	10 active healthy men age 20 ± 2 years	1. 60-min running downhill at a −10% gradient + 40-min run on the treadmill at a 1% gradient at 65% VO_2_max in a temp. of 33 °C with 50% RH 2. 60-min flat run + 40-min run on the treadmill at a 1% gradient at 65% VO_2_max in a temp. of 33 °C with 50% RH	pNGAL uNGAL uNGLA/u.f.
Mellor et al. (2013) [42]	22 subjects age 36 ± 2.4 years (7 females, 15 males)	ascent from sea level to 1085 m over 6 h	pNGAL
Sugama et al. (2013) [59]	14 male triathletes age 28.7 ± 7.9 years	duathlon race: 5 km of running + 40 km of cycling + 5 km of running	uIL-6 uIL-8
Kanda et al. (2014) [37]	9 untrained healthy men age 24.8 ± 1.3 years	One leg calf-raise exercise 10 sets of 40 repetitions of exercise at 0.5 Hz with 3 min rest between sets	pNGAL uNGAL uFABP
Hewing et al. (2015) [24]	167 recreational runners age 50.3 ± 11.4 years (89 females, 78 males)	marathon	sCyst-C
Andreazzoli et al. (2017) [41]	18 professional male cyclists age 31.5 ± 4 years	mountain stage of one of the major European professional cycling competitions	pNGAL uNGAL
Mansour et al. (2017) [46]	22 heathy amateur runners age 44 (range 22–63) years (13 females, 9 males)	marathon	uNGAL uKIM-1 uIL-6, uIL-8, uIL-18, uTNFα, uYKL-40, uMCP-1
Bongers et al. (2017) [44]	60 marchers age 29 ± 78 years (30 females, 30 males)	30, 40 or 50 km for three consecutive days	uCyst-C, uNGAL, uNGAL/uCyst-C, uNGAL/Cr, uNGAL/uOsm uKIM-1, uKIM-1/uCyst-C, uKIM-1/uCr, uKIM-1/uOsm
Bongers et al. (2018) [26]	35 active healthy males age 23 ± 3 years	150-min cycle ergometer test at 80% of HRmax until 3% hypohydration (samples taken after 30 and 50 min)	sCyst-C, uCyst-C uNGAL, uNGAL/uCyst-C, uNGAL/uCr, uNGAL/uOsm uKIM-1, uKIM/uCyst-C, uKIM-1/uCr, uKIM-1/uOsm
Spada et al. (2018) [48]	58 healthy volunteers age 24 (range 21–28) years (29 males, 29 females)	4 min of HIIRT	uNGAL, uNGAL/uCr uIL-18, uIL-18/uCr uCalbindin, uCalbindin/uCr uTTF, uTTF/uCr
Wołyniec et al. (2018) [47]	19 healthy amateur runners age 35.74 ± 6.99 years (9 females, 10 males)	treadmill run test	uNGAL, uNGAL/uCr uKIM-1, uKIM-1/uCr
McDermott et al. (2018) [39]	40 healthy cyclists age 52 ± 9 years	endurance cycling event (5.7 ± 1.2 h) in heat (33.2 ± 5.0 °C, 38.4 ± 10.7% RH)	sNGAL
Chapman et al. (2019) [18]	12 healthy adults age 24 ± 5 years (3 females, 9 males)	4 h exercise in heat (35,1 °C, 61% RH)	pNGAL uNGAL, uNGAL/u.f.
Wolyniec et al. (2019) [45]	16 Healthy amateur runners age 36.7 ± 8.2 years (2 females, 14 males)	10- and 100-km runs	uCyst-C uNGAL, uNGAL/uCr uKIM-1, uKIM-1/uCr
Jouffroy et al. (2019) [51]	47 healthy males age 43 ± 7 years	80-km ultramarathon	uNGAL, uNGAL/uCr uKIM-1, uKIM-1/uCr
Poussel et al. (2020) [27]	24 healthy runners age 36.5 (range 24–57) years (1 female, 23 males)	120-km ultramarathon with 5700 m of positive elevation gain	sCyst-C uNGAL, uNGAL/uCr
Chapman et al. (2020) [43]	13 healthy adults age 23 ± 2 years (3 females, 10 males)	2 h exercise in a heat (temp 39 °C, 32 % RH)	uNGAL uIGFBP-7 uTIMP-2
Kosaki et al. (2020) [53]	116 adults without chronic kidney disease age 62 (range 24–83) years (31 females, 85 males)	incremental short maximal exercise using a cycling ergometer	uL-FABP/uCr
Semen et al. (2020) [50]	54 healthy runners age 47 ± 15 years (21 females, 33 males)	half marathon after use of 400 mg single-dose ibuprofen: two groups: 1. supplemented with MOF-VVPP 2. Control	uNGAL, uCyst-C uIL-6, uIL-8, uIL-18, uTNFα
Semen et al. (2020) [49]	1. 35 runners age 44 ± 2 years (17 females, 18 males) 2. 45 runners age 55 ± 2 years (24 females, 21 males)	1. 10 km run 2. half marathon	uNGAL

**Abbreviations:** u—urinary, s—serum, p—plasma, Cyst-C—cystatin C, NGAL—neutrophil gelatinase-associated lipocalin, KIM-1—kidney injury molecule-1, L-FABP—liver-type fatty-acid-binding protein, Il—interleukin, TTF3—trefoil factor-3, TNFα—tumor necrosis factor α, YKL-40—chitinase 3-like protein 1, MCP-1—monocyte chemoattractant protein-1, IGFBP-7—insulin-like growth factor binding protein 7, TIMP-2—tissue inhibitor of metalloproteinases-2, Cr—creatinine, Osm—osmolality, u.f.—urine flow, uMarker/uCyst-C—urinary marker normalized to cystatin C, uMarker/uCr—urinary marker normalized to creatinine, uMarker/uOsm—urinary marker normalized to osmolality, uMarker/u.f.—urinary marker normalized to urine flow, HIIRT—high-intensity interval resistance training, VO_2_max—maximal oxygen consumption, HRmax—maximal heart rate, RH—relative humidity, monomeric and oligomeric flavanols (MOF-VVPP).

## Abbreviations

AKI	acute kidney injury
u	urinary
s	serum
p	plasma
NGAL	neutrophil gelatinase-associated lipocalin
KIM-1	kidney injury molecule-1
Cyst-C	cystatin C
Cr	creatinine
L-FABP	liver-type fatty-acid-binding protein
IL	interleukin
TTF3	trefoil factor-3
TNFα	tumor necrosis factor α
YKL-40	chitinase 3-like protein 1
MCP-1	monocyte chemoattractant protein-1
IGFBP7	insulin-like growth factor binding protein 7
TIMP-2	tissue inhibitor of metalloproteinases-2
uMarker/uCyst-C	urinary marker normalized to cystatin C
uMarker/uCr	urinary marker normalized to creatinine
uMarker/uOsm	urinary marker normalized to osmolality
uMarker/u.f.	urinary marker normalized to urine flow
eGFR	estimated glomerular filtration rate
CKD EPI	Chronic Kidney Disease Epidemiology Collaboration equation
HIIRT	high-intensity interval resistance training
VO_2_max	maximal oxygen consumption
HRmax	maximal heart rate
RH	relative humidity
OA	osteoarthiritis
MOF-VVPP	monomeric and oligomeric flavanols
VO_2max_	maximal oxygen consumption
MMP-9	matrix metallopeptidase 9
